# Supplementary material for: Systematic Review of Primary Outcome Measurements for Chronic Fatigue Syndrome/Myalgic Encephalomyelitis (CFS/ME) in Randomized Controlled Trials
Source: J Clin Med. 2020 Oct 28;9(11):3463. doi: 10.3390/jcm9113463 (PMC7692998; doi:10.3390/jcm9113463)
Supplement: Supplementary file 1 [file jcm-09-03463-s001.pdf]

Supplementary Table S1. Summary of RCTs included in this review

Study [Ref] (year)	N. of participant (female)	Intervention	Diagnostic criteria	[C] Primary measurement	Cut off score (range of score)
Jung et al. [41] (2019)	97 (60)	Myelophil	1994 Fukuda	CFQ-11 (Total score)	-
Montoya et al. [32] (2018)	128 (92)	KPAX002	1994 Fukuda	CIS (total score)	-
Clark et al. [21] (2017)	211 (167)	Guided graded exercise self-help	NICE, 2003 Reeves & 1991 Sharpe	CFQ-11 (total score)<br>SF-36 (physical function)	-
Janse et al. [34] (2017)	240 (145)	Internet based CBT	1994 Fukuda & 2003 Reeves	CIS (fatigue severity)	CIS: 35 (7-56)
Nilsson et al. [47] (2017)	62 (52)	(-)-OSU6162	1994 Fukuda & 2011 Carruthers	CGI<br>MFS (total score)	-
Roerink et al. [35] (2017)	50 (50)	Anakinra	1994 Fukuda & 2003 Reeves	CIS (fatigue severity)	CIS: 40 (7-56)
Friedberg et al. [59] (2016)	137 (121)	Fatigue self-management	1994 Fukuda	FSS (total score)	-
Ostojic et al. [57] (2016)	21 (21)	Guanidinoacetic acid	1994 Fukuda	MFI (general fatigue)	-
Park et al. [53] (2016)	78 (61)	Human placental extract	1994 Fukuda	FSS (total score)<br>MFI (total score)<br>VAS (fatigue)	-
Windthorst et al. [54] (2016)	28 (28)	HRV biofeedback + GET	1994 Fukuda	MFI (total score)	-
Arnold et al. [58] (2015)	60 (52)	Duloxetine	1994 Fukuda	MFI (general fatigue)	MFI: 13 (4-20)
Castro-Marrero et al. [66] (2015)	73 (73)	CoQ10 + NADH	1994 Fukuda	FIS (total score)	-
Kim et al. [60] (2015)	150 (98)	Acupuncture	1994 Fukuda	FSS (total score)	-
Pinxsterhuis et al. [22] (2015)	137 (121)	Group based self-management	1994 Fukuda & 2003 Carruthers	SF-36 (physical function)	-
Vos-Vromans et al. [17] (2015)	122 (97)	CBT, MRT	1994 Fukuda	SF-36 (total score)<br>CIS (fatigue severity)	CIS: 40 (7-56)
Wiborg et al. [23] (2015)	204 (157)	Group CBT	1994 Fukuda	SF-36 (physical function)<br>CIS (fatigue severity)<br>SIP-8 (total score)	CIS: 35 (7-56)<br>SIP: 700 (0-5799)
[A] Sulheim et al. [67] (2014)	120 (86)	Clonidine hydrochloride	1994 Fukuda	N. of steps per day	-
Montoya et al. [55] (2013)	30 (20)	Valganciclovir	1994 Fukuda	MFI (total score)	-
Ng & Yui [42] (2013)	99 (68)	Acupuncture	1994 Fukuda	CFQ (total score)	-
[A] Nijhof et al. [36] (2012)	135 (111)	Internet based CBT	1994 Fukuda	CIS (fatigue severity)	CIS: 40 (7-56)

Tummers et al. [30] (2012)	123 (96)	Guided self-instruction	1994 Fukuda & 2003 Reeves	CHQ-CF (physical function)<br>SAR<br>SF-36 (physical + social function)<br>CIS (fatigue severity)	CHQ-CF: 85 (0-100)<br>SAR: 85 (0-100)<br>SF-36: 70 (0-100)<br>CIS: 35 (7-56)
Young [65] (2012)	26 (25)	Lisdexamfetamine dimesylate	1994 Fukuda	BRIEF-A (total score)	[B] BREIF-A: 65 (t-score)
Burgess et al. [24] (2011)	80 (63)	CBT	1991 Sharpe & 1994 Fukuda	SF-36 (physical function)<br>CFQ-11 (total score)	-
Fluge et al. [62] (2011)	30 (21)	Rituximab	1994 Fukuda	VAS (symptom severity)	-
Núñez et al. [18] (2011)	115 (101)	CBT + GET	1994 Fukuda	SF-36 (total score)	-
Rimes & Wingrove [43] (2011)	35 (29)	Mindfulness based cognitive therapy	1991 Sharpe or 1994 Fukuda	CFQ-11 (total score)	CFQ: 4 (0-11)
White et al. [7] (2011)	640 (495)	APT, CBT, GET	1991 Sharpe	SF-36 (physical function)<br>CFQ-11 (total score)	SF-36: 60 (0-100)<br>CFQ: 6 (0-11)
[A] Chalder et al. [63] (2010)	63 (43)	Family-focused CBT	1991 Sharpe or 1994 Fukuda	SAR	-
The et al. [37] (2010)	67 (47)	Ondansetron	1994 Fukuda	CIS (fatigue severity)<br>SIP-8 (total score)	CIS: 35 (7-56)<br>SIP: 800 (0-5799)
Tummers et al. [25] (2010)	169 (133)	Stepped care	1994 Fukuda	SF-36 (physical function)<br>CIS (fatigue severity)<br>SIP-8 (total score)	CIS: 35 (7-56)<br>SIP: 700 (0-5799)
Wearden et al. [26] (2010)	296 (230)	Pragmatic rehabilitation + supportive listening	1991 Sharpe	SF-36 (physical function)<br>CFQ-11 (total score)	SF-36: 70 (0-100)<br>CFQ: 4 (0-11)
Hobday et al. [27] (2008)	52 (43)	Low sugar and yeast diet	1994 Fukuda	SF-36 (physical function)<br>CFQ (total score)	-
Walach et al. [31] (2008)	409 (308)	Distant healing	1991 Sharpe or 1994 Fukuda	SF-36 (mental health summary)	-
The et al. [38] (2007)	57 (39)	Acclydine	1994 Fukuda	CIS (fatigue severity)<br>SIP-8 (total score)	CIS: 35 (7-56)<br>SIP: 800 (0-5799)
Blockmans et al. [33] (2006)	60 (45)	Methylphenidate	1994 Fukuda	CIS (total score, concentration)<br>VAS (fatigue, concentration)	-
McDermott et al. [46] (2006)	71 (51)	BioBran MGN-3	1994 Fukuda	CFQ-11 (physical score)	-
O'Dowd [19] (2006)	153 (102)	Group CBT	1994 Fukuda	SF-36 (total score)	-
Moss-Morris et al. [48] (2005)	49 (34)	GET	1994 Fukuda	CGI	-
[A] Stulemeijer et al. [28] (2005)	69 (62)	CBT	1994 Fukuda	SF-36 (physical function)<br>CIS (fatigue severity)<br>SAR	-

Blacker et al. [49] (2004)	434 (285)	Galantamine hydrobromide	1994 Fukuda	CGI	-
Weatherley-Jones et al. [56] (2004)	103 (61)	Homeopathic treatment	1991 Sharpe	MFI (total score)	-
Olson et al. [61] (2003)	20 (13)	Dexamphetamine	1994 Fukuda	FSS (total score)	-
Brouwers et al. [39] (2002)	53 (37)	Polynutrient supplement	1994 Fukuda	CIS (fatigue severity)<br>SIP-8 (total score)<br>APS (CDC checklist)	CIS: 40 (7-56)<br>SIP: 750 (0-5799)
Zachrisson et al. [50] (2002)	100 (100)	Staphypan Berna	1994 Fukuda	CPRS-15 (total score)<br>CGI	-
Powell et al. [29] (2001)	148 (116)	Education of GET	1991 Sharpe	SF-36 (physical function)	SF-36: 25 (0-30)
Prins et al. [40] (2001)	270 (212)	CBT	1994 Fukuda	CIS (fatigue severity)<br>SIP-8 (total score)	CIS: 40 (7-56)<br>SIP: 800 (0-5799)
Rowe et al. [51] (2001)	100 (66)	Fludrocortisone acetate	1994 Fukuda	CGI	-
Cleare et al. [44] (1999)	32 (20)	Hydrocortisone	1991 Sharpe & 1994 Fukuda	CFQ-11 (total score)<br>CGI	-
Forsyth et al. [64] (1999)	26 (17)	NADH	1994 Fukuda	APS (CDC symptoms)	-
Peterson et al. [20] (1998)	25 (19)	Fludrocortisone	1988 Holmes & 1994 Fukuda	SF-36 (total score)<br>VAS (symptom severity)	-
Wearden et al. [45] (1998)	136 (97)	Fluoxetine + GET	1991 Sharpe	CFQ (total score)	CFQ: 4 (0-14)
Fulcher & White [52] (1997)	66 (49)	GET	1991 Sharpe	CGI	-

[A] These RCTs were conducted with adolescent participants.
[B] Scores lesser than 1.5 standard deviations above the standardized population mean (t-score 65) were excluded.
[C] Twenty-one RCTs used the multiple tools as the primary measurement.
APS: author produced scale, APT: adaptive pacing therapy, BRIEF-A: Behavior Rating Inventory of Executive Function–Adult version, CBT: cognitive behavioral therapy, CFQ: Chalder Fatigue Questionnaire, CGI: Clinical Global Impression, CHQ-CF: Child Health Questionnaire-Child Form, CIS: Checklist Individual Strength, CPRS-15: Comprehensive Psychopathological Rating Scale-15, FIS: Fatigue Impact Scale, FSS: Fatigue Severity Scale, GET: graded exercise therapy, HRV: heart rate variability, MFI: Multidimensional Fatigue Inventory, MFS: Mental Fatigue Scale, MRT: multidisciplinary rehabilitation treatment, SAR: school attendance rate, SF-36: 36-item Short Form health survey, SIP-8: Sickness Impact Profile-8, VAS: Visual Analogue Scale.

Supplementary Table S2. General characteristics of self-reported survey measurements used in RCTs

Scale (N. of item)	Development purpose (published year)	Subscales (N. of item)	Scoring method (range of total score)
SF-36 (36)	General health status (1988)	physical function (10), general health (5), mental health (5), role function/physical (4), vitality (4), role function/emotional (3), social function (2), pain (2), health change (1)	3,5,6-point scale, Y/N (0-100) Higher = better
CIS (20)	Fatigue in CFS (1994)	fatigue severity (8), concentration (5), motivation (4), physical activity (3)	7-point scale (20-140) Higher = worse
CFQ (14)	Fatigue (1993)	physical symptom (8), mental symptom (6)	4-point scale or Y/N (0-42 or 0-14) Higher = worse
CGI (1)	Severity of mental disorder (1976)	No subscale division	7-point scale (1-7) Higher = worse
MFI (20)	Fatigue in cancer (1995)	general fatigue (4), mental fatigue (4), physical fatigue (4), reduced activity (4), reduced motivation (4)	5-point scale (20-100) Higher = worse
SIP-8 (86)	Health status (1975)	social interactions (20), ambulation (12), alertness behavior (10), home management (10), mobility (10), work (9), recreation & pastimes (8), sleep & rest (7)	Y/N (0-5799) Higher = worse
FSS (9)	Fatigue in MS & SLE (1989)	No subscale division	7-point scale (9-63) Higher = worse
VAS (not fixed)	Psychological feature (1921)	No subscale division	0-10 graphical rating (0-10) Higher = worse
APS (8, 50)	Symptom severity in CFS (1999, 2002)	No subscale division	APS1: Y/N (0-8) Higher = worse APS2: 4-point scale (50-200) Higher = worse
BRIEF-A (75)	Executive function behavior (2000)	emotional control (10), plan/organize (10), initiate working (8), inhibit (8), organization of material (8), working memory (8), self-monitor (6), task monitor (6), validity (6), shift (5)	3-point scale (t-score) Higher = worse
CHQ-CF (87)	Health-related quality of life in children (1990)	general behavior (17), mental health (16), self-esteem (14), general health perception (12), physical function (9), family activity (6), role function/behavior (3), role function/physical (3), role function/emotional (3), bodily pain (2), change in health (1), family cohesion (1)	4,5,6-point scale (0-100) Higher = better
CPRS-15 (15)	Severity of psychiatric symptoms (1978)	No subscale division	7-point scale (0-90) Higher = worse
FIS (40)	Fatigue (1994)	psychosocial function (20), cognitive function (10), physical function (10)	5-point scale (0-160) Higher = worse

MFS (15)	Mental fatigue in neurological diseases (2010)	No subscale division	7-point scale (0-45) Higher = worse

BRIEF-A: Behavior Rating Inventory of Executive Function–Adult version, CFQ: Chalder Fatigue Questionnaire, CGI: Clinical Global Impression, CHQ-CF: Child Health Questionnaire-Child Form, CIS: Checklist Individual Strength, CPRS-15: Comprehensive Psychopathological Rating Scale-15, FIS: Fatigue Impact Scale, FSS: Fatigue Severity Scale, MFI: Multidimensional Fatigue Inventory, MFS: Mental Fatigue Scale, MS: multiple sclerosis, SF-36: 36-item Short Form health survey, SIP-8: Sickness Impact Profile-8, SLE: systemic lupus erythematosus.
